# Master-Slave Control Scheme in Electric Vehicle Smart Charging Infrastructure

**DOI:** 10.1155/2014/462312

**Published:** 2014-05-26

**Authors:** Ching-Yen Chung, Joshua Chynoweth, Chi-Cheng Chu, Rajit Gadh

**Affiliations:** Department of Mechanical and Aerospace Engineering, University of California at Los Angeles, 420 Westwood Plaza, Los Angeles, CA 90095-1594, USA

## Abstract

WINSmartEV is a software based plug-in electric vehicle (PEV) monitoring, control, and management system. It not only incorporates intelligence at every level so that charge scheduling can avoid grid bottlenecks, but it also multiplies the number of PEVs that can be plugged into a single circuit. This paper proposes, designs, and executes many upgrades to WINSmartEV. These upgrades include new hardware that makes the level 1 and level 2 chargers faster, more robust, and more scalable. It includes algorithms that provide a more optimal charge scheduling for the level 2 (EVSE) and an enhanced vehicle monitoring/identification module (VMM) system that can automatically identify PEVs and authorize charging.

## 1. Introduction

Every plug-in electric vehicle (PEV) that is purchased instead of a dedicated fossil fuel burner is a good step in the direction of energy independence and lower greenhouse gas emissions. As ever more of these PEVs hit the road, sufficient charging infrastructure becomes ever more important in furthering the proliferation of PEVs in the car market. In order to maximize the charging infrastructure that can be installed on a given electrical grid, optimization needs to not only account for energy production, but also account for constraints that may appear in the system at any level. PEVs not only burden the energy production system, but also pockets of PEVs in certain areas may strain the local grid and transformers. Furthermore, each new current electric vehicle supply equipment (EVSE) requires a dedicated electrical circuit that incurs expenses that limit the number of EVSEs that will be installed. WINSmartEV [1–3] is a software based PEV monitoring, control and management system that not only incorporates intelligence at every level so that charge scheduling can avoid grid bottlenecks, but it also multiplies the number of PEVs that can be plugged into a single circuit. This combination of optimizing the use of the electrical grid while multiplying the number of PEVs per circuit is a one-two punch against the limits of the electrical infrastructure in charging PEVs.

Simple commercial charging stations such as Leviton and ClipperCreek, which simply provide basic charging function without network features, do not provide network services for smart charging purposes. One exception is Coulomb Inc. Coulomb devised its own proprietary network-controlled charging system through a remote server [4, 5], but these stations are not suitable for current sharing purposes because they only have one or two outlets. A method of electrical circuit sharing for charging stations is proposed by Coulomb [6]; however, no details of charging algorithms are provided.

Since WINSmartEV is software based, intelligent charging algorithms can be implemented and updated when needed. The algorithms can be developed based on user's time, energy price, or energy amount. A charging algorithm that relies on a smart phone interface for entering PEV data, such as arrival and departure times and initial and final state of charge (SOC), is proposed in [7]. The scheduling algorithm proposed in [8] requires the initial energy states of a PEV as the input. These approaches are not valid unless the user provides the actual SOC data. To solve this problem, the authors of [9] propose a custom-built module, named vehicle monitoring/identification module (VMM), which reads the in-vehicle controller area network (CAN) data bus and transmits SOC data via a ZigBee wireless link to a charging station and then onto the charging controller. However, without insider knowledge of the PEV manufacturers, identifying data location on the CAN bus could present a challenge for obtaining the SOC data. Several charging algorithms are presented in [10–12]; however, none of them mentioned a method to achieve variable current and multiplexing control, let alone the collaboration between the control center and the charging stations.

In order to fully utilize the power resource on the local grid, collaboration between the master controller (server) and the slave controllers (charging stations) in the PEV charging infrastructure is required to manage the charging sessions and/or control the current to the PEVs. This ability is not incorporated into the current WINSmartEV design. In this paper, a master-slave control scheme for the electric vehicle smart charging infrastructure is proposed to enhance the performance and features of this smart charging infrastructure. These improvements include hardware upgrades that will enable better collaboration between EVSE and server, enhanced smart charging algorithms, improved safety requirements, and incorporating RFID authentication and authorization into the VMM system. This paper is structured in the following way. First the current version of WINSmartEV is outlined in Section 2. Next, the proposed upgrades to the smart charging control scheme is discussed in 2 sections, Section 3 for level 1 EVSEs and Section 4 for level 2 EVSEs. Then an RFID authentication and authorization scheme is discussed in Section 5.

## 2. Existing WINSmartEV Infrastructure

**Figure 1:**
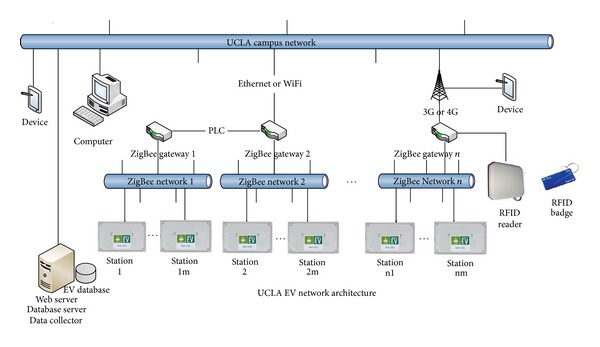
Network architecture of WINSmartEV.

There are three subsystems in UCLA WINSmartEV smart charging infrastructure including the control system, the communication system, and the metering system. Some special features such as smart charging algorithms [13], safety requirement integration [14], and RFID mesh network system for user authentication and authorization [15] are developed based on the existing hardware and software.  Figure 1 shows the network architecture of WINSmartEV.

**Figure 2:**
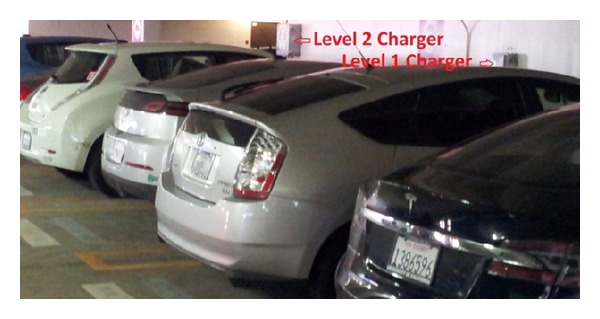
Installation of smart charging stations.

In order to implement the electrical power sharing concept, a four-outlet smart charging station connected to a single circuit is designed and implemented in [16]. The one-circuit-to-four-outlet design is based on the limitation of normal circuit installation (30 A continuous) and the minimum PEV charging current (6 A) defined in J1772 standard. Theoretically, the number of outlets could be 5 in order to fully utilize the maximum capacity of the circuit. However, in real practice, it will easily trip the circuit breaker if any one of five PEVs draws a bit more than specified current.  Figure 2 shows the installation of a level 2 smart charging station and a level 1 smart charging station in a UCLA parking lot.

The details of the subsystems including control system, communication system, and metering system are described in the subsections.

### 2.1. Control System

In this section, the functionality of master controller (central server), the slave controller (charging stations) is described in the following. There are two types of charging stations, level 1 charging stations that connect to standard 120 V household circuits and level 2 charging stations that connect to 208 V or 240 V circuits for faster charging. The level 1 charger controls four EVSmartPlug outlets to provide power to the PEVs. Because the PEV user plugs the PEV's trickle charging cable into the outlet to charge PEV, the control system switches the outlet on and off in order to control the 120 V power to the trickle charging cable.

The level 1 charging stations are currently controlled by a server-based central controller equipped with smart scheduling algorithms. Different algorithms, including real time algorithms and scheduling algorithms, can be implemented to control charging. Round-robin and FCFS (first-come, first-served) are examples of real-time algorithms. Scheduling algorithms can be developed to include many factors such as time, energy price, energy amount, or SOC. For example, a round-robin algorithm, which only turns on 1 channel and charges 1 PEV at a time, is currently used to schedule charging in the level 1 EVSmartPlug station to share a single 120 V power source with four PEVs. This algorithm only takes into account how long the PEVs have been present. As more sophisticated algorithms are developed, the central controller has the flexibility and extensibility to be updated to include these new algorithms.

Because it incorporates J1772 standards with cables that can be plugged directly into the PEVs, the level 2 charger is required to turn on and off the power to the PEVs by controlling relays and rate the power that each PEV pulls by controlling the duty cycle of the pilot signal. The hardware and firmware of the level 2 smart charging station local controller is designed and implemented in [16].

In order to accelerate the response of the smart charging stations by reducing the traffic between the smart PEV charger and the control center, a power information collector (PIC) in [17] is designed to collect the power information locally and relay it to the control center periodically. The response time can be further reduced by pushing the information to the control center; thus, a fast response smart PEV charging infrastructure is achieved. However, because the control scheme is server-based, the server will need to wait for *T*_wait_ due to the communication delay and the response time of the charging station and the PEV. In order to accelerate the performance of the system, a master-slave control scheme is required as proposed in Section 3.

### 2.2. Communication System

In [16], there is a multiple protocol gateway inside the smart charging station to provide communication services for multiple functions. To connect to the internet, there are three types of methods including 3G, Ethernet, and WiFi. 3G communication is required due to its flexibility and accessibility to be everywhere as long as the cellular signal exists, especially where wired or WiFi communication is unavailable. When using Ethernet for communication, the gateway can directly connect to the internet with a static IP or a dynamic IP assigned from a DHCP router. When Ethernet connection and 3G service are unavailable in a parking area, WIFI or PLC can be used to connect to another gateway or router that does have an internet connection. The EVSE's gateway can use a PLC module on its Ethernet port to connect to other gateways or routers connected to any electrical circuit on the same transformer. When using WiFi for local communication, the gateway needs to be setup as a client to connect to other gateways or routers. In this case, a port forwarding method is used on the other gateway or router so that the server can access the client gateway.

The information interchanged between the gateway, meters, and the control unit are through ZigBee communication. The function of the ZigBee coordinator on the gateway is to handle the messages between the gateway and the end devices or routers including the meters, control units, and vehicle monitoring/identification modules (VMMs) [9] on the PEVs. In order to dispatch the commands and parameters to the desired devices, the ZigBee coordinator needs to recognize and register the unique MAC addresses of the end devices or routers. Since a number of devices communicate using ZigBee mesh network capabilities, only one gateway is required in a geographic locale.

The current system has two types of controllers, one with and the other without ZigBee communication. The controller without a ZigBee module talks to the gateway directly through USB port with RS232 communication. On the other hand, the controller set with ZigBee communication consists of ZigBee coordinator and ZigBee end-device. The gateway talks to the ZigBee coordinator to dispatch or receive response from the ZigBee end-device. Both types of controllers require a RS232-USB adapter cable in between the gateway and controllers. In order to ensure proper functionality, the RS232-USB adapter cable must be compatible with the gateway. When the 3G dongle is used for communication, both the 3G dongle and RS232-USB adapter cable must be assigned a USB port and only the assigned USB ports should be used.

### 2.3. Metering System

**Figure 3:**
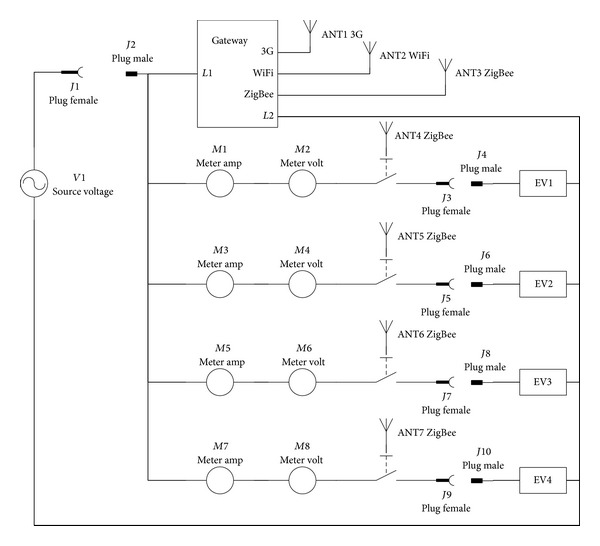
Schematics of metering system.

The metering system in both level 1 and level 2 charging stations consists of a gateway and four meters. The meter inside the charging station returns its power information, including voltage, current, frequency, power factor, and energy consumption, to the gateway upon receiving the command of power information retrieval through ZigBee communication. The meters need to join the ZigBee mesh network created by the ZigBee coordinator embedded in the gateway. The function of the metering system also requires the association of the meters' ID and the physical outlet numbers. The detailed schematics of a four-outlet metering system are shown in Figure 3.

## 3. Proposed Control Scheme and Results for Level 1 EVSE

The processes involving the collaboration between the server and the charging stations such as the smart charging algorithm [13], safety requirement [14], and the RFID authentication and authorization [15] are the prior arts in publications. In order to fully utilize the power resource on local grid and improve the performance of the PEV charging infrastructure in the management of charging sessions or current control, the collaboration between the master (server) and the slave (local controller) is required. Therefore, a master-slave control scheme for the electric vehicle smart charging infrastructure is proposed to enhance the performance of the features including smart charging algorithms, safety integration, and RFID authentication and authorization. The proposed control scheme involves a server-based central controller and local controllers inside the charging stations. The details of the collaboration scheme for the level 1 EVSE is presented in this section and the details for the level 2 EVSE will be presented in the next section. RFID authentication and authorization will be discussed in Section 5.

### 3.1. Local Controller Design

**Figure 4:**
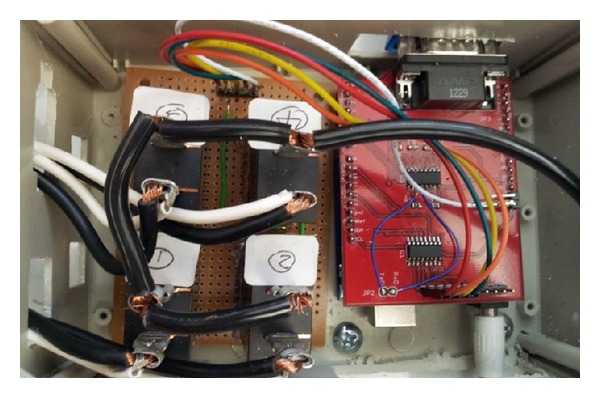
Local controller with controllability over power to outlets.

In current level 1 charging station design [1–3], there is no local controller inside the charging station. When the data pull method sends a power information request command from the server to a charging station, the signal must pass through the internet and through a 3G network before it reaches the gateway of the charging station. Then the gateway relays the power information request command to the specific meter it is meant for. When the gateway receives a reply from the meter, it relays the response back to the server where the information travels back in reverse order. With multiple meters requiring multiple requests each, the aggregated round trip times cause slow performance. In order to enhance the system's performance and shorten the response time of the system, a device named the power information collector (PIC) [17] collects the power information locally in order to send it in to the server together as one packet. By decreasing the number of communications required for status reports and control operations, the PIC significantly decreases the delay time for switching PEV charging sessions or changing current to the PEVs. In order to accelerate the response time of the charging station, based on the design of the PIC, a local controller with the controllability over the power on the outlets is implemented as shown in Figure 4.

Because the local controller is responsible for turning on/off the outlet, it reduces the round trips of command and response between the server and the charging stations. Thus, it accelerates the response time of the charging station. Since the power information is available on the local charging station, local charging algorithms can be realized on this design. Certain charging algorithms can be implemented at the local level. With less communication traffic between the server and the charging stations, the charging algorithms at the local level could be more efficient than that at the server level. By reducing the traffic between the server and the charging stations, the improvements allow the control center to serve a larger system, which enhances the capability of the existing WINSmartEV framework.

Nevertheless, considering the calculation power of the microprocessor, only certain simple charging algorithms, such as round-robin and Schedule Time, can be implemented in the microprocessor. Thus, with local charging algorithms implemented on the charging station level, the server will only need to select the mode of the charging algorithms of each charging station. This will save significant server computing resources. With local charging algorithms implemented, the control center could handle a larger smart charging system due to the reduction of traffic between the control center and the smart charging stations.

However, because of the lack of computing power at the local level, more complex charging and scheduling algorithms still need to be implemented on the server. No matter where the charging algorithm sits, two major operation flows including ENABLE CHARGING and DISABLE CHARGING involve in the smart charging algorithms at server level. In each operation flow, two subprocesses including READ OUTLET ON/OFF STATUS and READ POWER INFORMATION need to be done.

This smart charging infrastructure can use its metering system to monitor the simple commercial charging stations such as Leviton, ClipperCreek, and Schneider so that the server can have control over these charging stations. These charging stations can run switching type charging algorithms at the server level including a round-robin algorithm or a fair charging algorithm [13]. In order to have fair usage of power resource for every PEV user, currently, a round-robin algorithm is used to schedule charging in the multiplex charging system WINSmartEV. In current practice, the round-robin algorithm is developed at the server level for the lack of local controller.

### 3.2. Fair Charging Algorithm

In order to appeal to more users, a fair charging algorithm [12] is proposed to maximize fairness in the allocation of charge time for the smart plug charger. The fair charging algorithm is designed for a switching type of charging stations such as the EVSmartPlug, where only one PEV can charge at a time. In this case, each user's charge ratio *τ* is defined as the ratio of the charging time *T*_Charge_ and the stay time *T*_Stay_ in

(1) $$\begin{array}{c} \tau \equiv \frac{T_{\text{Charge}}}{T_{\text{Stay}}}. \end{array}$$

The fairness of the charging system depends on how close all the users' charge ratios *τ* are to each other. The fairness system can also be stated as follows: for a given charging event, every user's mean charge ratio *μ*(*τ*) should be close to the mean charge ratio of every other user charging at the time *μ*[*μ*(*τ*)]. Therefore, both *σ*[*μ*(*τ*)] and *μ*[*σ*(*τ*)] must approach 0 as the system approaches complete fairness. The fairness index *α* is defined in (2) to indicate the fairness of the system:

(2) $$\begin{array}{c} \alpha \equiv 1-\frac{\left\{\sigma \left[\mu \left(\tau \right)\right]+\mu \left[\sigma \left(\tau \right)\right]\right\}}{2}. \end{array}$$

Even though the round-robin algorithm seems fair, the fairness index [13] shows that it favors the first user that starts charging over the users that arrive later. In the fair charging algorithm, when the second user's charging session overlaps that of the first user's charging session, the server predicts the second user's charge time and the first user's stay time in order to create a fair charging schedule. The schedule will allot each of the two PEVs the charge time required so that they leave with same charge time ratio. The schedule will switch charging enough to avoid the risk of a large charge imbalance, but not so much as to take up too much time switching. When a third or a fourth PEV arrives, then new fair charge schedules are created that takes into account the new user. If the time to switch charging between PEVs is close to zero, then the optimization algorithm can be executed. The fairness could be maximized by continuously switching charging power between PEVs. However, there is a noticeable time delay in switching charging sessions between PEVs due to data pull method. Thus, the period of time to switch charging from one PEV to the next can be as high as minutes, given hardware and network constraints. If the system switched continuously between users, much charge time would be wasted in the switching process, causing all users to be worse off. Fairness maximization can be obtained while only switching charging once if exact stay time of the PEV is known. If PEV's stay time is unknown, fewer switching may often leave the charge time for each PEV lopsided and unfair. Therefore, the optimization of the fairness algorithm needs to take into account both the confidence of stay time and the time wasted in switching. Therefore, the Fair Charging Algorithm counts heavily on the accuracy of the prediction of user's stay time. A forecast of users to the PEV charging station in [18] can be used for more accuracy on user's stay time. If the prediction of the user's stay time is accurate, fairness maximization can be obtained while only switching charging once. There is no way for the charging infrastructure to retrieve a PEV's SOC status for the purpose of user's stay hour prediction unless extra devices, VMM [9], for SOC data retrieval are equipped on the PEV. The PEV's SOC status should not be considered available by using the current PEV charging station standard J1772. Therefore, based on a user's historical charging records, for predictable people, either *u*(*T*_Stay_) or a linear regression function is used to predict *T*_Stay_ of the user. For unpredictable people, the average stay time of all users *u*⌊*u*(*T*_Stay_)⌋ is used for prediction. A number of switches may be required if the prediction of the stay time is not accurate enough.

Currently, the fair charging algorithm is implemented at server level. In order to have better performance, the calculations which rely on the historical data in the data base should still be finished at the server level. The local controller inside the charging station is responsible to execute the charging schedule calculated by the server. After the server calculates the charging schedule according to the selected charging algorithm, it sends the charging schedule to the stations. The charging stations control the charging sessions based on the schedule. When some charging events happen during the charging sessions, the local controller requests the server to update the charging schedule. When the server receives the request of charging schedule change, it calculates a new charging schedule for the charging stations.

### 3.3. Safety Requirement

Because the control of pilot signal for the level 1 charging station, EVSmartPlug, takes place within the PEV's trickle charge cable, the automatic reset of GFCI is not required by UL certification. Therefore, a single commercial breaker with GFCI on the power source fulfills all the safety requirements.

## 4. Proposed Control Scheme and Results for Level 2 EVSE

The possible efficient control scheme of smart charging algorithms can be developed based on user preference and the local power capacity. The central controller, or server, equipped with smart charging algorithms sends commands or schedule to the charging station through a multiple protocol gateway. The central server functions as the master controller while the local controllers, embedded in the charging station, serve as the slave controller in the infrastructure.

### 4.1. Local Controller Design

**Figure 5:**
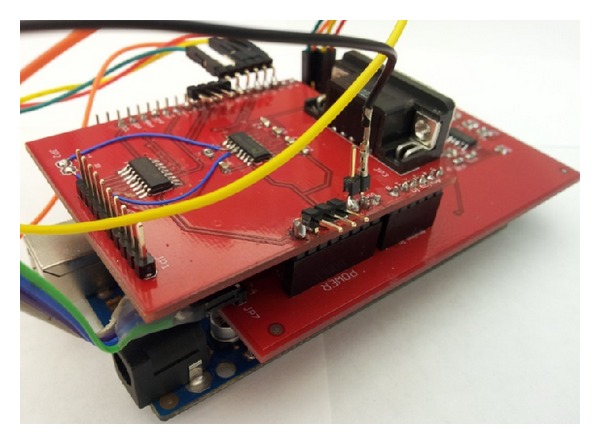
Simplified J1772 EVSE controller without ZigBee function. (1) RS232 module. (2) Pilot signal generator/monitor. (3) Aduino Mega2560.

The current level 2 chargers have a ZigBee-based local (slave) controller with multiple functions including the pilot signal generator, pilot signal monitor, safety relay controller, and autoreset function as implemented in [16]. In this design, three microprocessors are utilized to fulfill the functionality. In the current practice, because each charging station is equipped with a multiple protocol gateway, the information exchange between the charging stations can be fulfilled by WiFi or Ethernet. Therefore, the ZigBee function on the local controller is indeed a redundant communication channel. In order to simplify the design and enhance the features and functionalities, the ZigBee module has been removed and a more powerful microprocessor, ATMega2560, with more input and output pins (I/Os) has been added as shown in Figure 5. The pilot signals are created by ATMega2560's internal timer and monitored by its analog input pins. In this design, only one microprocessor is used to fulfill the aforementioned functionality.

**Figure 6:**
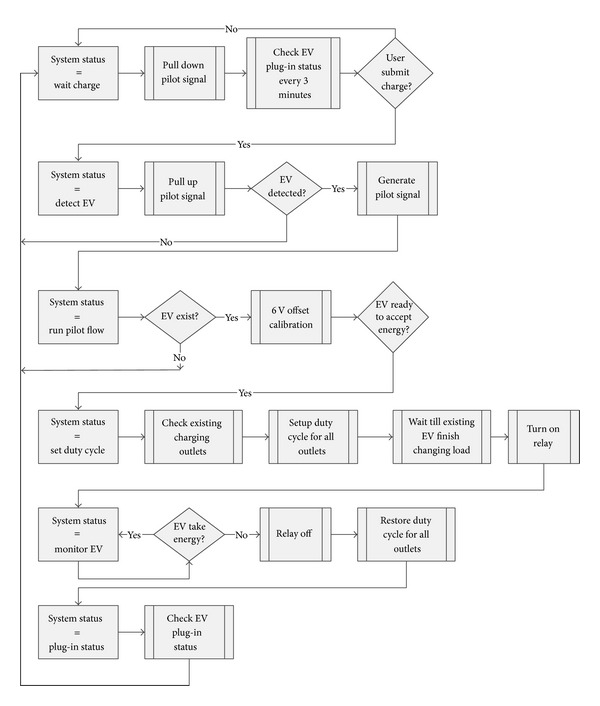
State machine of simple Current Sharing Algorithm.

With enhanced processing power, simple charge scheduling algorithms can be implemented on local device. These simple current sharing algorithms can be designed and implemented by revising the firmware-based state machine as shown in Figure 6. In the simple Current Sharing Algorithm, the local controller assigns the available power to the designated outlet by setting up the duty cycle of the pilot signal before the Monitor EV stage.

**Figure 7:**
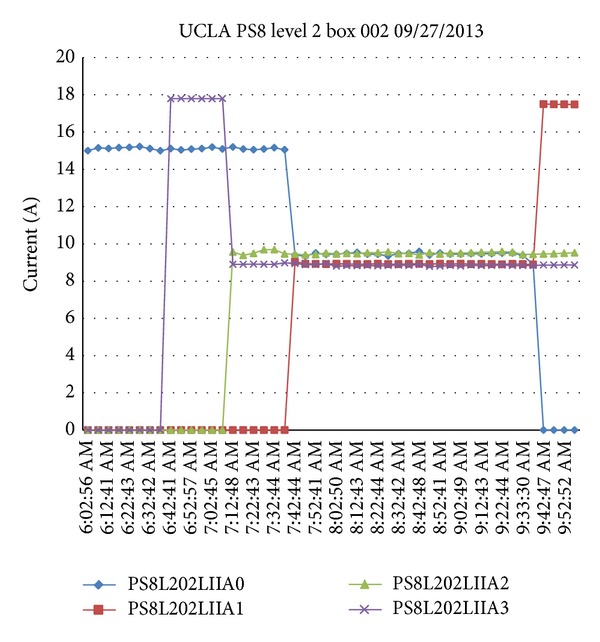
Example of simple current sharing algorithm.

The process of setting up the duty cycle is inserted in between the processes of Run Pilot Flow and Monitor EV. The current sharing algorithm is based on the configuration of the box. If there is no PEV charging in an adjacent channel, the firmware will set the maximum available current to the given outlet. Otherwise, the firmware will divide the current for the PEVs to share. Note that experimental results have shown a five-second delay in the PEV response time [16]. Once a PEV is unplugged, the local controller restores the power to other PEVs. In [16], the Monitor EV stage is handled periodically based on the timer interrupt flag. In order to fulfill the J1772 standard to handle a faster PEV unplug detection, the Monitor EV stage is moved to the main loop for continuously checking unplug status.  Figure 7 shows an example configuration of a simple Current Sharing Algorithm. In this configuration, Ch1 and Ch2 share one power circuit while Ch3 and Ch4 share another power circuit.

The firmware sets up the maximum duty cycle 30% (18 A) for Ch1 initially. When the adjacent channel Ch2 is plugged in, the maximum duty cycle is divided by 2 and becomes 15% (9 A). When the PEV at Ch1 is fully charged or unplugged, the duty cycle of Ch2 is set back to the maximum duty cycle. Ch3 and Ch4 present the same result.

**Figure 8:**
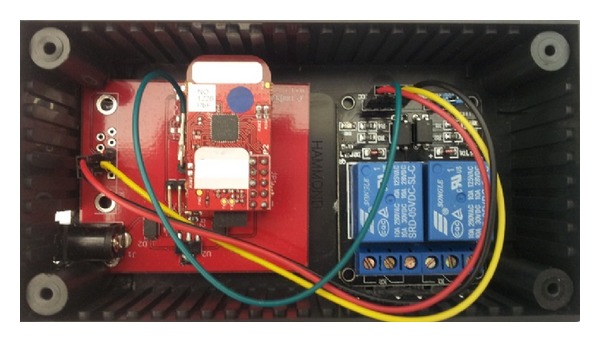
ZigBee-based local controller for ClipperCreek charging station.

Simple commercial charging stations can also be modified as smart charging stations by connecting a metering system and local controllers. In the ClipperCreek case, the charging station model CS-40 provides the terminals for three stage-current control (30 A, 6 A, and 0 A) [19]. To control the connectivity of the terminals, a ZigBee-based local controller with relay module is designed and implemented as shown in Figure 8.

With ZigBee communication, local charging stations can exchange information with each other in a local area. With this design, only one gateway is required per locale; thus, it saves in communication costs.

In order to implement a variable continuous current control on the simple commercial charging stations, an extra circuit would need to be installed in between the charging station and the PEV to emulate the behavior of the PEV. The design requirements for this circuit would include the ability to generate a pilot signal with variable duty cycle in response to the command from the server. The design of the extra circuit, which is beyond the scope of this paper, is not addressed here.

### 4.2. Fair Current Sharing Algorithms

To involve the smart charging algorithms at server level, the server can select or disable local charging algorithms embedded in the firmware of the local controller. No matter where the charging algorithm sits, three major operation flows at server level need to be engaged including ENABLE CHARGING, DISABLE CHARGING, and PILOT SIGNAL DUTY CYCLE CHANGE. In each operational flow, there are three subprocesses including READ METER ON/OFF STATUS, READ METER'S POWER INFORMATION, and READ OUTLET'S STATUS. The power information includes the voltage, current, and active power. The outlet's status includes pilot signal's duty cycle, safety relay on/off status, PEV plug-in status, and firmware-based state machine's stage.

To deal with a variable current charging station, a fair current sharing algorithm is proposed. Considering a variable current control charging station, the fairness index is now related to the energy consumption *E* during the stay time *T*_Stay_, which equals the average power *P* for the user in

(3) $$\begin{array}{c} P\equiv \frac{E}{T_{\text{Stay}}}=V\left(t\right)I\left(t\right)\times \frac{t}{T_{\text{Stay}}}. \end{array}$$

For a completely fair system, the average power *P* of each user should be the same, which means each user's charge rate is the same during the stay time. For a fair enough system, each user's charge rate should be close enough. Assuming *V*(*t*) is a constant, a current share ratio *ρ* can be defined in

(4) $$\begin{array}{c} \rho \equiv \frac{I\left(t\right)\times t}{I_{\text{MAX}}\times T_{\text{Stay}}}, \end{array}$$

where *I*_MAX_ is the current capacity.

The time sharing type of fair charging algorithm required for switching chargers can now be viewed as a special case of a current sharing algorithm with a discrete current instead of a variable current. If the system is fair, every user's *μ*(*ρ*) should be close to *μ*[*μ*(*ρ*)]. Therefore, both *σ*[*μ*(*ρ*)] and *μ*[*μ*(*ρ*)] approach 0 if and only if the system approaches complete fairness for each user. Here, a new fairness index *β* is defined in

(5) $$\begin{array}{c} \beta \equiv 1-\frac{\left\{\sigma \left[\mu \left(\rho \right)\right]+\mu \left[\sigma \left(\rho \right)\right]\right\}}{2}. \end{array}$$

The fairness index *β* approaches 1 if and only if both *σ*[*μ*(*ρ*)] and *μ*[*μ*(*ρ*)] approach 0, which is used to indicate the fairness of the system. The parameter *σ*[*σ*(*ρ*)] is viewed as the convergence of the system; *σ*[*σ*(*ρ*)] converges to 0 when the system is fair.

**Figure 9:**
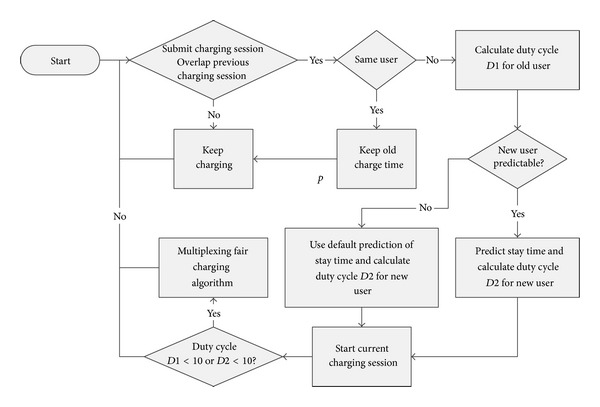
Fair Current Sharing Algorithm.

Figure 9 shows the flow of Fair Current Sharing Algorithm.

When the second user's charging session overlaps that of the first user, the server still predicts the second user's charging time and the first user's stay time. However, instead of calculating charge time allocation for each PEV, the server calculates the maximum current each PEV is allowed to draw current *I* based on the remaining energy consumption and the current share ratio *ρ* in (6). Instead of switching charging between the users' charging sessions, the server communicates the current *I* that each PEV is allowed by changing the duty cycles *D* of the pilot signal:

(6) Ii=Di×0.6I1,i=(ρTarget×IMAX×T1,Stay−I1,i−1×t)(T1,Stay−t)I2,i=ρTarget×IMAX.

*I*_1,*i*_ and *I*_2,*i*_ represent the maximum allowed current for the first and second user, respectively. *ρ*_Target_ is the target value of the current share ratio of the system. The third user's session is treated as an overlap of the second user's session, and the forth user's turn is treated as an overlap of the third user's session. Note that, in practical implementation, the maximum allowed current drawn is a discontinuous function of *D*_*i*_ based on J1772 standards:

(7) Ii=0, 0<Di<10Ii=0.6×Di, 10≤Di<85Ii=2.5×(Di−64), 85≤Di<96Ii=0, Di≥96.

Because 240 V with 30 A is the most common installation, only the conditions in (8) are taken into account:

(8) Ii=0, 0<Di<10Ii=0.6×Di, 10≤Di<30.

If the result of maximum current drawn *I*_*i*_ is less than 6 A, the Fair Charging Algorithm with a 6 A maximum will be used instead of the Current Sharing Algorithm.

Similar to the Fair Charging Algorithm proposed in [13], the Fair Current Sharing Algorithm also counts on the accuracy of the prediction of user's stay time because the duty cycle calculation in (6) is based on the predicted stay time.

The command sets for the server and return values from the charging station are summarized in [16]. Based on the experiments in [9, 16], the server waiting time for the command sets in [15, 16] is formulized and summarized in Table 1.

In order to accelerate the server's performance, the server's waiting time should be set to different values according to the command set sent from Table 1. In the case of duty cycle change, to shorten the server's waiting time *T*_waiting_, it can be set to be variable values based on *I*_init_ and *I*_final_ rather than a fixed value to satisfy all conditions. Moreover, the simulation of pilot signal duty cycle change in [16] shows that it takes 30 ms to reach steady state. This value needs to be compensated in the firmware of the local controller.

The authors in [18] concluded that charging algorithms with power information retrieval can be implemented locally in the charging station. The system with embedded charging algorithms, in which the traffic between the charging station and control center is reduced, is faster than that with remote charging algorithms which are implemented on the server. However, from the experimental result, the Fair Current Sharing Algorithm performs better than the Simple Current Sharing Algorithm with more users' information. Because the Fair Current Sharing Algorithm is based on the user's historical charging records, it is more proper to implement it on the server. With the charging stations equipped with PICs set on data pushing mode, the Fair Current Sharing Algorithm becomes more practical due to the improved response time of the system caused by the PIC.

Nevertheless, it is also possible to obtain the user's stay time, if the user's PEV is equipped with the VMM proposed in [9], which facilitates using the battery's state of charging (SOC) to predict the user's stay time. In this way, it is possible to implement the Fair Current Sharing Algorithm in the charging station.

### 4.3. Safety Requirement

As for the safety requirement for the level 2 charging station, since the charging station controls PEV charging by the pilot signal, the charging station is required to handle the GFCI function in both J1772 and UL standard. Although the authors in [20] claim the GFCI of a networked charging station can be reset remotely, no details of control methods or schematics are presented. To provide a safe smart technology for charging PEVs, a design for the safety system is presented in [14], which is implemented on all levels of control. The administrator can turn the relays of the charging stations on or off and check their status by sending out commands. The charging stations can be reset manually or automatically on schedule as long as the connection between the server and the charging stations exists. The pilot signal monitor can reset the whole system by turning off the switch on the power source of the charging station upon receiving the system reset command from the server. After the charging station loses power, the switch on the power source of the charging station is back to its normal position such that the charging station turns on again. Any emergency action taken at the top level will have a delay time that depends on the condition of the wireless communication including 3G, WiFi, ZigBee, and Cloud. Thus, a fast acting local unit is implemented to stop charging in case of an emergency.

In order to prevent electrical hazards, there should be no voltage on the handle of the charging cable until it is plugged into a PEV. The detection of the PEV plug-in status is implemented in the state machine of the firmware of the control unit based on the J1772 standard. The voltage of the pilot signal pin on the handle should be +12 V when there is no PEV connected to the charging station. After plugging in the PEV, the voltage will be +9 V or +6 V depending on whether or not the PEV is ready to accept energy. The PEV plug-in status detection is implemented in the state machine in the firmware of the control unit. Furthermore, the charging station is required to shut off the power immediately to prevent the hazard of electric shock when there is an abnormal diversion of current from one of the hot wires. The ground fault circuit interrupter (GFCI) detects the difference of current between two hot wires and shuts off the safety relay when the difference has crossed the threshold amperage. Unlike a traditional GFCI which requires manually pressing the reset button, a pure hardware GFCI with a remote reset function is used to increase the reliability. The power to the PEV can be controlled by the server, the control unit of the charging station, and the GFCI circuit.

**Figure 10:**
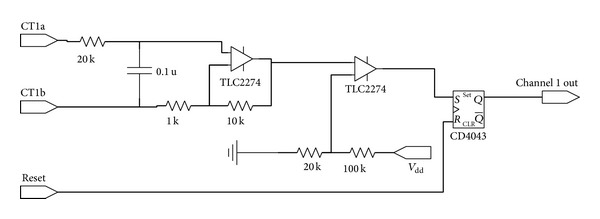
Schematic of GFCI.

In [14], one leg of the contactor is controlled by the pilot signal monitor, while the other leg is controlled by the GFCI. The pilot signal monitor can reset the GFCI by toggling the switch on the power source of the GFCI. However, in real world applications, in order to have independent control over each GFCI channel, instead of controlling the power source of the GFCI board, the microprocessor generates the reset signal for the SR latch as shown in Figure 10.

Every time when one outlet is tripped, the microprocessor is able to reset the GFCI independently after the user unplugs the PEV without affecting other PEV's charging session.

**Figure 11:**
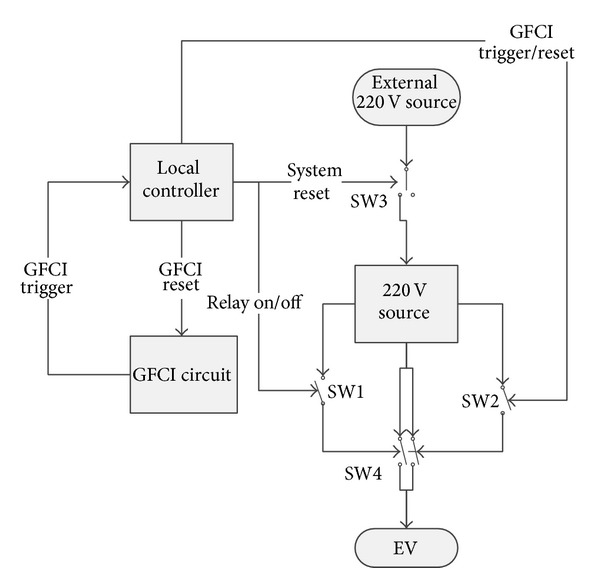
Schematic of safety control for the relay.

Because the GFCI board is sensitive, false alarms are easily triggered due to the glitch at the rising edge in the output signal of GFCI board. In order to avoid triggering false alarms, the local controller deglitches the output from GFCI board and shuts off the contactor.  Figure 11 shows the new relay control method to avoid GFCI false alarms.

**Figure 12:**
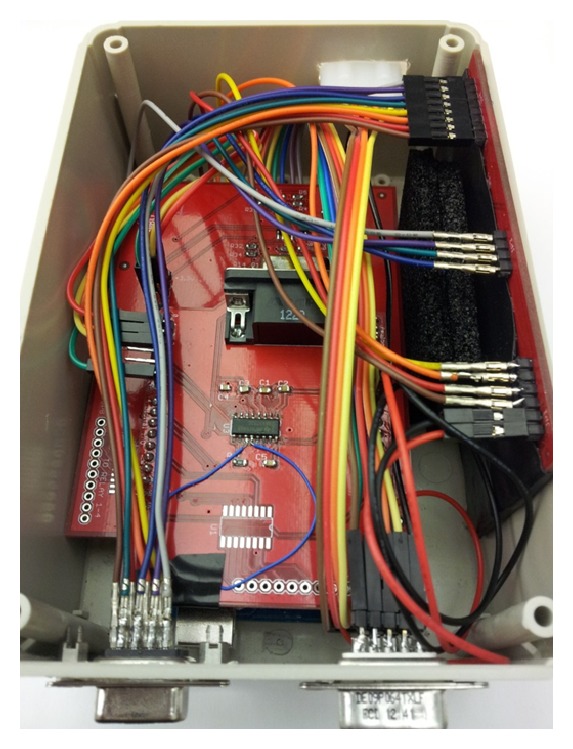
Local controller for level 2 charging station with GFCI.

In the new design, the GFCI board feeds its output to the local controller. Instead of controlling one leg of the contactor directly by GFCI board, the local controller controls both legs of the contactor for different conditions. The implementation of Non-ZigBee Level 2 J1772 local controller with GFCI function is shown in Figure 12.

**Figure 13:**
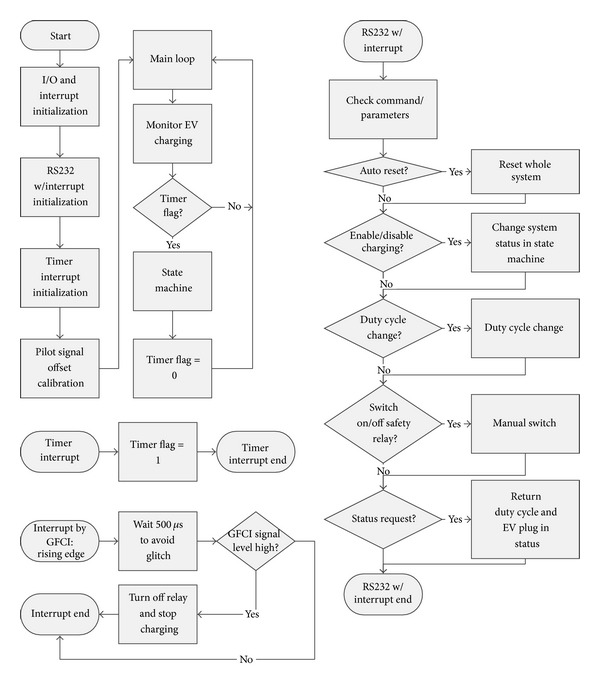
Local controller firmware flow chart.

In order to have the fastest response time, the interaction between the GFCI board and the microprocessor is handled by the interrupt routine in the firmware as shown in Figure 13.

In the implementation of the GFCI function, four interrupt pins are used to monitor the outputs from the GFCI board with a rising edge trigger. In order to avoid the false alarms caused by the glitch at the rising edge, after one interrupt trigger, the outputs of the GFCI board are monitored by the digital input pins with 500 us delay in the interrupt loop. If the controller detects that the output pin is HIGH, it terminates the power to the PEV by turning off the specific relays, thereby shutting off the contactor.

The GFCI of the outlet will be reset after the user unplugs the PEV. When the GFCI of the outlet is triggered, the system status of the state machine for the outlet will go to UNPLUG CHECK, in which the local controller keeps monitoring the unplug status until the user unplugs the PEV.

The design of GFCI in [14] is triggered by 14 mA difference between two hot wires on the positive cycle. Since the design of GFCI only works for positive cycle of the AC, if an abnormal diversion of current from a hot wire happens on the negative cycle, the GFCI trigger will be delayed by 8.3 ms, which is half cycle of 60 Hz. In addition, the GFCI circuit itself has approximately 1 ms delay. In our proposed GFCI design, compared to the maximum delay time of 8.3ms after the GFCI triggers, which is half cycle of 60 Hz, a 500 us delay time is acceptable. To satisfy the safety requirement by UL (Underwriters Laboratories) certification, the total delay of GFCI function should meet the requirement of the maximum value, which is 24.9 ms as per UL standard. Overall, the maximum delay in our proposed GFCI is 9.8 ms, which satisfies the safety requirement by UL standard.

Besides the time delay requirement for GFCI, the UL requires extra circuits such as GFCI tester and voltage monitor on the contactors to fulfill the safety requirement. The GFCI tester is the circuit to test the GFCI function before energizing the contactor. A solution for the GFCI tester is to add an extra wire in the current sensor from 12 V dc source. Then, the local controller turns on a specified small current on this wire by using a digital output with a FET transistor and a power resistor. The voltage detector will check the circuit to see if the contactor is welded before enabling the contactor. If the contactor is welded, which means the charging station cannot stop power to the outlet, the system should stop providing service. A possible solution is to use a voltage divider with power resistors to obtain small AC voltage. Then, a transformer is inserted in between to isolate the AC and DC voltage. Later, the local controller detects this DC voltage with a Schmitt trigger through its digital input pin to see if there is voltage on the outlet of the charging station.

As mentioned before, because the GFCI board is sensitive, in order to reduce the magnetic field disturbance from the electromagnetic relay, the GFCI board needs to be enclosed in a grounded metal surface box.

Once the safety feature is certified, the UL does not allow firmware change by checking the CRC code of the firmware. Since the safety function is not charging all the time, in order to have the flexibility to add new features in the future, the separation of safety feature from the other functions is needed. Therefore, one possible solution is to user extra microprocessor to handle the safety features while the original one deals with other features. Thus, the charging station can keep updating with new features while satisfying the UL certification.

## 5. RFID Authentication and Authorization Scheme

In current WINSmartEV system, users are able to authenticate themselves through a mobile app [1–3]. A concept of mesh network radio frequency identification (RFID) charging authorization system in [15], which facilitates the authentication process at a smart charging station, allows charging authorization to take place at the moment of PEV arrival without user involvement. The mesh network provides robust connections between PEVs and charging stations in a real world environment subject to signal blocking conditions. The ZigBee routers in the VMMs serve as RFID tags while the ZigBee coordinator, attached to the Gateway in the charging station, serves as the RFID reader. The unique 64-bit MAC address of each ZigBee device is utilized as an RFID tag. The charging authentication process includes ZigBee MAC address retrieval, user authorization, and PEV plug-in status detection. When the charging station detects the PEV at a distance, the received signal strength indication (RSSI) of the handshake serves as the metric for identifying a PEV approaching a charging station. The PEV plug-in status detection is used to identify the presence of a PEV at a charging station and to associate the vehicle's ID with a particular channel. The mesh network RFID is developed based on existing hardware without additional cost and provides traditional RFID benefits while adding mesh network capability.

In order to add authorization/identification capability, the firmware of the ZigBee coordinator inside the charging station and the software on the server need to be redesigned. Rough processes in PEV charging authentication via RFID, including ZigBee MAC address retrieval, user authorization, and PEV plug-in status detection, are presented in [15]. However, more details of the collaboration between the master controller (server) and the local controller (ZigBee coordinator) need to be addressed for implementation. The details of the proposed master-slave control scheme for RFID authentication and authorization are presented in the following.

In [15], the authentication and authorization processes are periodically handled by the server. In the authentication process, new PEV arrivals are checked by the ZigBee coordinator inside the charging station. The server sends out the “rgst” command to check if new tag IDs have been registered after RFID reader initialization. The “stat” command is later sent out to identify which charging station a newly arrived PEV is plugged into.

In real practice, in order to accelerate the performance of the system, the system needs to be modified to data push system. Instead of periodically sending “rgst” command to retrieve the new tag IDs, the ZigBee coordinator pushes tag IDs to the database once new tag ID is detected. Notice that the local controller inside the charging station serves the trigger signal of PEV plug-in status. Once the PEV plug-in status is detected, the local controller pushes the status to the database. In the authorization process, if the tag ID corresponds to an authorized user account in the database, the command to enable charging is sent out to begin PEV charging.

In the collaboration between master and slave controllers in RFID mesh network feature, several issues including handshake request interval, PEV approaching and leaving determination, and exception condition handling are discussed in the following.

About the handshake request interval, the maximum time for a two-hop response is 2 seconds in [9], which means *T*_ZigBee_ has maximum value of 2 seconds. Considering *T*_CAN_read_ is with maximum value of 0.1 second, *T*_wait_ will have to be greater than 2.1 seconds per equation (1). As a result, 2.1 second minimum waiting interval must be incorporated on the local controller. Therefore, an interval much larger than 2.1 seconds needs to be incorporated for detecting an approaching PEV. Taking 3G communication delay presented in [10] into account, the maximum round trip time of 3G is around 5 seconds, which means the server will need to wait 7.1 seconds to receive a response to a data request.

As for PEV approaching and leaving determination, in most cases, the accepted speed limit in parking lot is 5 mph, which means a PEV approaches a charging station by 4.5 meters every 2 seconds. Assuming that the PEV parks 5 meters away from the charging station, after a PEV is detected at a distance of 50 meters, the station will have a maximum of 10 handshakes to determine whether the PEV is approaching or leaving.

Considering the exception condition handling, when more than two PEVs come to the same charging station around the same time, the charging station might not have a way to associate the IDs with the corresponding outlets. In this case, the server needs an exception handling process to handle the charging sessions. If the arriving PEVs have different size of on-board chargers, the charging station is able to associate the IDs with outlets due to different current when charging. However, if the on-board chargers are the same size, the server cannot associate IDs with the outlets. In this case, the server can later associate the charging sessions with IDs and outlets when the PEVs leave by detecting the PEVs' RSSI; the server can also associate the IDs and outlets by SOC when PEVs are fully charged before they leave. If the PEVs with same size on board chargers arrive and leave around the same time without being fully charged, there is no need to distinguish the charging session because their drivers will be billed for the same energy consumption.

## 6. Conclusion

In this paper, we have proposed, designed, and implemented a master-slave control scheme for the PEV smart charging infrastructure. This scheme includes adding a power information collector (PIC) to the level one EVSE that not only makes it faster and more scalable, but also it enables the level 1 charger to execute operations within the EVSE itself. With these features, the level 1 EVSE can execute simple charging algorithms such as round-robin locally. Furthermore, these enhanced capabilities allow the server to control the level 1 charger as a slave, making the network structure more robust.

The hardware for level 2 EVSE has been updated in order to simplify the design and enhance its features and functionalities. This update includes removing the redundant ZigBee communication system and updating the microprocessor to a more powerful one. These updates allow the implementation of a power sharing algorithm locally. This is the simplest algorithm for level 2 charging. Furthermore, a fair charging algorithm appropriate for the level 2 EVSE is proposed. This charging algorithm is calculated on the server side and executed with minimal instructions on the EVSE side. Enhancements to the GFCI system have also been proposed that runs a system check to ensure that the GFCI system is operating properly and will shut off power when required in order to prevent hazards.

Algorithms have also been proposed to enhance the capability of the VMM system. These enhancements will include the capability to automatically authenticate and authorize each PEV as it approaches the EVSE. This allows the user to drive up to the EVSE and connect the cable. These algorithms control the system to automatically do the rest, so the user does not have to log into the server to begin charging.

Every PEV that is purchased instead of a dedicated fossil fuel burner is a good step in the direction of energy independence and lower greenhouse gas emissions. As ever more of these PEVs hit the road, sufficient charging infrastructure becomes even more important in proliferating PEVs throughout the car market. With its new enhanced ability to multiply the number of PEVs serviced for a given electrical infrastructure, WINSmartEV is poised to not only serve as a part of the nationwide smart grid system but as part of the larger push for PEV proliferation.

## Figures and Tables

**Table 1:** Waiting time of command sets.

Comd.	Description and waiting time
atrs	Auto-reset the charging station
	*T*_Waiting_ > 0.5*T*_3G_ + *T*_GatewayStartUp_

duty	Change the duty cycle of pilot signal
	$T_{\text{Waiting}}>\{\begin{array}{cc} 1000+400\times I_{\text{final}}-0.5\times T_{\text{3G}}, & I_{\text{init}}=0\\ 110-0.5\times T_{\text{3G}}, & I_{\text{final}}=0\\ 310-0.5\times T_{\text{3G}}, & I_{\text{init}}<I_{\text{final}}\\ 5060-0.5\times T_{\text{3G}}, & I_{\text{init}}>I_{\text{final}} \end{array}$

enab	Enable EV charging
	*T*_Waiting_ > *T*_3G_ + *T*_EnableChargingProcess_ + *T*_EVResp_

rely	Turn on/off relay manually
	*T*_Waiting_ > *T*_3G_

rest	Disable EV charging
	*T*_Waiting_ > *T*_3G_

resp	ZigBee handshake response
	*T*_Waiting_ = 0

rgst	Return all registered ZigBee MAC address
	*T*_Waiting_ > *T*_3G_ + *T*_GatewayUSBTimeout_

stat	Charging station status request
	*T*_Waiting_ > *T*_3G_ + *T*_GatewayUSBTimeout_

test	ZigBee handshake request
	*T*_Waiting_ = 0
